# Circular RNAs: Potential Applications as Therapeutic Targets and Biomarkers in Breast Cancer

**DOI:** 10.3390/ncrna7010002

**Published:** 2021-01-05

**Authors:** Debina Sarkar, Sarah D. Diermeier

**Affiliations:** Department of Biochemistry, University of Otago, Dunedin 9016, New Zealand; debina.sarkar@otago.ac.nz

**Keywords:** circular RNA (circRNA), non-coding RNA, breast cancer, biomarker, therapeutic target

## Abstract

Circular RNAs (circRNAs) are a class of non-coding RNAs that form a covalently closed loop. A number of functions and mechanisms of action for circRNAs have been reported, including as miRNA sponge, exerting transcriptional and translational regulation, interacting with proteins, and coding for peptides. CircRNA dysregulation has also been implicated in many cancers, such as breast cancer. Their relatively high stability and presence in bodily fluids makes cancer-associated circRNAs promising candidates as a new biomarker. In this review, we summarize the research undertaken on circRNAs associated with breast cancer, discuss circRNAs as biomarkers, and present circRNA-based therapeutic approaches.

## 1. Introduction

Breast cancer (BC) is the most frequently diagnosed cancer and the leading cause of cancer death among females in over 100 countries [1]. According to the Global Cancer Statistics 2018, about 2.1 million females are newly diagnosed with the disease annually (24.2% of total cases), and more than 60,000 deaths are caused by BC (15.0%) [1]. In addition, incidence rates for BC have been rising in the last decade and have far exceeded those of other cancers in both developed and developing countries [2]. Breast cancer can be broadly classified into different subtypes based on the presence (+) or absence (-) of estrogen receptor (ER), progesterone receptor (PR), and human epidermal growth factor receptor 2 (HER2) [3]. Luminal subtypes are hormone receptor positive, whereas the HER2+ subtype is defined by overexpression of HER2 [3]. In triple negative breast cancer (TNBC), all three receptors are absent (ER−/PR−/HER2−) [4]. Compared to luminal and HER2 subtypes, TNBC is more invasive, has higher metastatic potential, and is associated with worse prognosis [4]. While some targeted therapies are available for luminal (endocrine therapy) [5] and HER2 (trastuzamab) subtypes [6], treatment options are limited for TNBC [7]. Currently, poly(ADP-ribose) polymerase inhibitors (PARPi) and immunotherapy (atezolizumab, pembrolizumab) are available for TNBC, but their application is restricted to a small subset of TNBC patients [7,8,9]. Overall, morbidity and mortality of BC remain high, in particular due to drug resistance and disease recurrence, indicating the need for new therapeutic targets and biomarkers [10].

Non-coding RNAs (ncRNAs) are a class of RNA molecules that have been well-demonstrated to play key roles in BC progression by impacting cell growth, metabolism, epithelial–to-mesenchymal transition (EMT), metastasis, or drug resistance [11]. A recent addition to the world of non-coding regulatory molecules is the class of circular RNAs (circRNAs). CircRNAs were originally discovered in 1976 as viroid RNA and were believed to be by-products of aberrant RNA splicing with little functional potential [12]. Recently, circRNAs have resurfaced and garnered attention due to the availability of high-throughput sequencing, with more than 183,000 circRNAs identified in humans thus far [13,14,15,16]. About 10% of all expressed genes (both coding and non-coding) are able to produce circRNAs [17]. They lack terminal 5′ caps and 3′ poly-A tails and are a stable and conserved class of RNA molecules [16,18]. CircRNAs are classified into three groups: exonic, intronic, and exon–intronic [19]. The expression of circRNAs is highly cell type- and tissue-specific [20,21], and some circRNAs are more abundant than their linear counterparts [22]. Additionally, no clear correlation has been detected between the abundance of circRNAs and their corresponding linear RNAs [20,22,23]. These observations suggest that circRNAs are likely functional and not mere by-products of RNA processing.

CircRNAs have been described to be associated with several diseases, including cancer [24], which lends support to examination of these molecules as functional moieties. Recently, circRNAs have garnered interest as potential novel diagnostic and prognostic biomarkers for BC due to their presence in many human bodily fluids, such as plasma and saliva [25]. Furthermore, circRNAs are highly enriched and stable in extracellular vesicles (EVs), particularly in tumor-derived EVs [26]. Thus, the potential functional roles of circRNA are becoming a novel focus in cancer research, with the aim of exploiting these transcripts as new biomarkers or therapeutic targets. This review summarizes recent advances in the field of circRNAs associated with BC progression, and discusses potential applications of circRNAs as biomarkers and therapeutic targets.

## 2. Biogenesis, Discovery, and Biological Functions of CircRNAs

### 2.1. Biogenesis of CircRNAs

**Figure 1:**
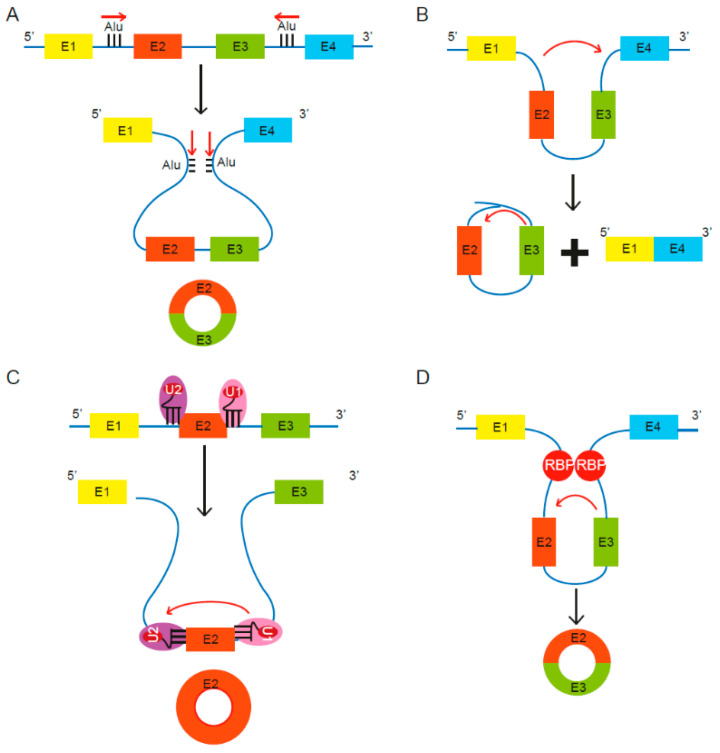
Proposed models of circular RNA (circRNA) biogenesis. Four proposed routes lead to the formation of circRNAs. (**A**) The pairing of intronic inverted repeat elements flanking either side of the exons leads to formation of circRNA [13]. (**B**) The alternative splicing mechanism “exon skipping” leads to an mRNA consisting of exons 1 and 4, as well as a lariat structure containing the skipped exons 2 and 3 [27]. (**C**) Under limited spliceosome activity, a shift from canonical splicing to back-splicing occurs that produces single-exon circRNAs, as cross-exon interactions are not replaced with cross-intron interactions [28]. (**D**) RNA binding proteins (RBPs) bind to recognition sites in introns near the circRNA-forming splice sites and promote circularization by bringing the circle-forming exons into close proximity [29,30].

CircRNA biogenesis can be broadly classified into two types: lariat driven or aided by either the spliceosomal machinery or RNA binding proteins (RBPs) (Figure 1).

Lariat driven circRNA biogenesis can occur via short, complementary repeat elements in flanking introns, which have been reported to be critical for circularization of intervening exons for some circRNAs [13] (Figure 1A). Alternatively, lariat-driven circRNA biogenesis can involve “exon skipping,” commonly associated with the splicing of pre-mRNA [27] (Figure 1B). In an exon-skipping event, a single pre-mRNA transcript can yield both a linear spliced mRNA and a circRNA comprising the skipped exons [27]. This process involves the generation of a lariat intermediate containing the excised exons, after which the introns in the lariat are removed [25], leading to a circRNA as well as a linear transcript devoid of the skipped exon(s) [31]. In canonical splicing, a 5′ splice site is recognized by U1 small nuclear ribonucleoprotein (snRNP), U2 snRNP binds the upstream polypyrimidine tract and branch point sequence, and cross-exon interactions are mediated by factors such as serine and arginine-rich (SR) proteins [28]. The cross-exon interactions are subsequently replaced with cross-intron interactions to enable complete assembly of the spliceosome and generation of linear RNA [28]. However, limited spliceosomal activity encourages a shift from canonical splicing to back-splicing that produces single-exon circRNAs, as cross-exon interactions are not replaced with cross-intron interactions (Figure 1C) [28]. Lastly, some studies revealed the involvement of certain RNA binding proteins (RBPs) in the process of RNA circularization [29,30] (Figure 1D). Splicing factors such as Quaking (QKI) and Muscleblind (Mbl) are known to facilitate and enhance circRNA production [29,30].

### 2.2. Discovery and Detection of CircRNAs

**Figure 2:**
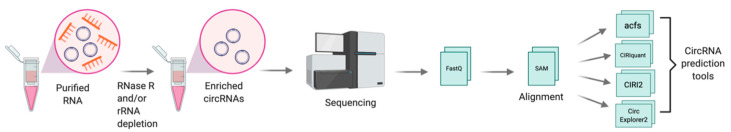
Schematic for discovery of circRNAs through RNA sequencing. Total RNA is isolated and treated with RNase R, in combination with or without depletion of ribosomal RNA (rRNA) and followed by RNA sequencing. Sequenced reads are quality assessed, trimmed and mapped to the human genome. High quality mapped reads are further processed through circRNA prediction tools such as acfs, [33], CIRIquant [34], CIRI2 [35], and CircExplorer2 [36]. Figure created with BioRender.com.

The first step towards the discovery of circRNAs involves the generation of non-polyadenylated transcriptomes. CircRNAs are enriched by depleting ribosomal RNA (rRNA) from total RNA, followed by an optional treatment with RNase R, a 3′-to-5′ exonuclease that preferentially digests linear RNAs, thereby allowing the enrichment of circular RNAs [32]. Libraries are prepared using a stranded total RNA library kit, coupled to paired-end high-throughput RNA sequencing (RNA-seq) [32]. Specific computational pipelines such as acfs [33], CIRIquant [34], CIRI2 [35], and CircExplorer2 [36] are designed to identify reads spanning the back-spliced junctions in reverse orientation (details outlined in Figure 2). To validate and quantify circRNAs in a targeted manner, qRT-PCR using primers designed to the back-spliced junctions can be applied [14].

### 2.3. Mechanism of Action of CircRNAs

**Figure 3:**
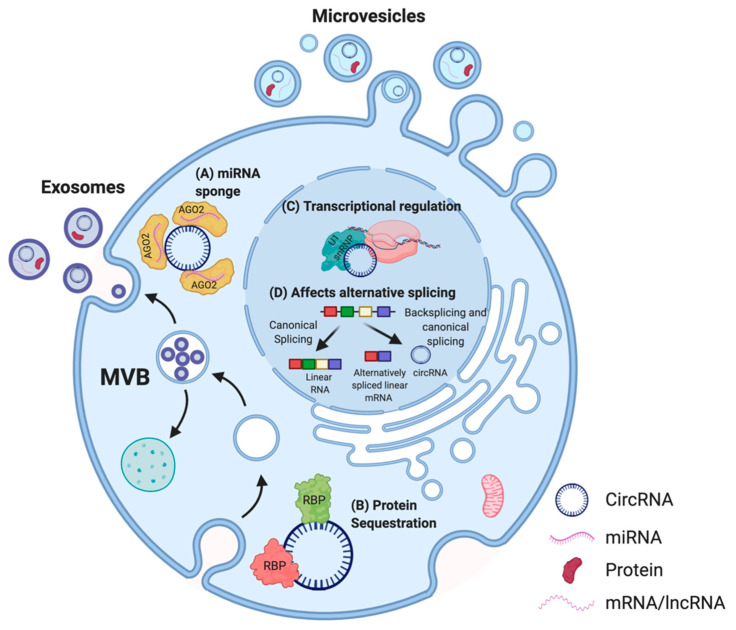
Proposed functions of circRNA in cells. In the cytoplasm, multiple circRNAs such as *CDR1as* have been shown to act as miRNA sponges (**A**) [38] whereas others possess binding sites for RBPs. For instance, *circFOXO3* can act as a protein (CDK2 and p21) sequestering molecule (**B**) [39]. In the nucleus, circRNAs are able to influence the transcription of their parental genes by interacting with the U1 small nuclear ribonucleoprotein particle (U1 snRNP) and RNA polymerase II (**C**) [40] and can affect alternative splicing of transcripts, thus altering gene expression (**D**) [41]. CircRNAs can also be secreted out via extracellular vesicles (EVs) such as microvesicles or exosomes, and enable cell–cell communication [42,43,44]. Adapted from “Extracellular Vesicle Separation by Density Gradient Ultracentrifugation,” by BioRender.com (2020). Retrieved from https://app.biorender.com/biorender-templates.

Evidence showing tissue-specific expression, high abundance, and stability has solidified the case for a functional potential of circRNAs in the cell [16]. Proposed functions of circRNAs are dependent on their sub-cellular localization in the cell [37] (Figure 3).

Thus far, the most commonly described and well-characterized function of circRNAs is their ability to act as micro RNA (miRNA) sponges. Multiple studies reported that circRNAs comprise binding sites for miRNAs, thereby acting as a molecular decoy and inhibiting the functions of those miRNAs on their mRNA targets [15,45,46,47]. This association between circRNAs and miRNAs has also been linked to several human diseases, including osteoarthritis [48], diabetes [49], neurodegenerative pathologies [38], and several cancers [50,51,52,53,54,55]. Other circRNAs were described to contain binding sites for RBPs, and may function as scaffolds to facilitate protein interactions, to regulate protein function, or to sequester the bound protein [56,57]. For instance, *circFOXO3*, a circRNA downregulated in cancer cells, forms an RNA–protein complex with cyclin-dependent kinase 2 (CDK2) and p21, which disrupts the interaction of CDK2 with cyclin A and cyclin E, and subsequently blocks cell cycle progression [39]. Thus, some circRNAs are able to sequester proteins in the cytoplasm and prevent their entry into the nucleus. In addition, circRNAs have been demonstrated to regulate the expression of their parental genes in a cis-acting manner [58]. *CircPAIP2* influences the transcription of its parental gene by interacting with the U1 snRNP and RNA polymerase II (RNA Pol II) (Figure 3) [40]. The binding of circRNAs to RNA Pol II can also affect alternative splicing [41]. A recent study also highlighted the biological significance of *circSCAR*, which localizes to mitochondria and may be a valuable therapeutic target for immunometabolic disorders [59]. Moreover, recent studies indicated that EV encapsulated synthetic or endogenous circRNAs can be transferred into acceptor cells, and these circRNAs retain their bioactivity after the transfer [42,43,44]. As numerous studies suggest that EVs play a crucial role in cell-to-cell communication [60], uncovering the roles of EV-associated circRNAs may be a useful addition to the development of new diagnostic and treatment approaches [61].

## 3. Function of CircRNAs in Breast Cancer

**Figure 4:**
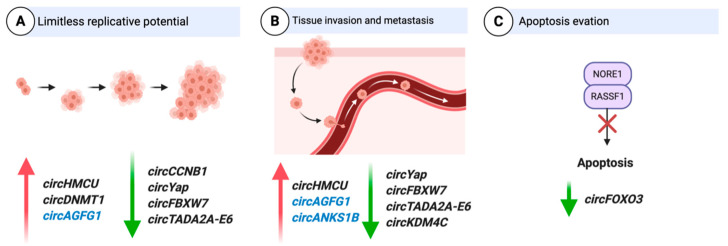
CircRNAs associated with hallmarks of breast cancer. Red arrows depict upregulation and green arrows depict downregulation. CircRNAs in blue are triple negative breast cancer (TNBC) specific. Adapted from “The Hallmarks of Cancer (Classical),” by BioRender.com (2020). Retrieved from https://app.biorender.com/biorender-templates.

A number of circRNAs have been implicated in the development of BC [52,62,63,64,65] and can be broadly categorized into two types: oncogenic circRNAs and tumor-suppressor circRNAs. Regardless of whether circRNAs act as promoters or inhibitors of BC progression, the mechanism generally impacts cell proliferation, invasion, apoptosis, and drug resistance. Some circRNAs have been well studied, and their function deciphered in regard to how they are involved in different hallmarks of BC such as proliferation, apoptosis, and activating invasion and metastasis [66]. Examples of circRNAs associated with various hallmarks of BC and with well-studied mechanisms of action are described below (Figure 4).

### 3.1. CircRNAs as Oncogenes

#### 3.1.1. Cell Cycle

***CircHMCU***: A gene expression omnibus (GEO) microarray dataset (GSE111504) obtained using parental MDA-MB-231 cells and corresponding lung metastatic cells (MDA-MB-231-LM2; LM2), was reanalyzed to identify circRNAs associated with BC metastasis [52]. *CircHMCU* was upregulated in LM2 cells compared to the parental MDA-MB-231 cells, and further validation using qRT-PCR-confirmed high *circHMCU* levels in BC tissues and correlation with poor prognosis [52]. Further studies revealed that *circHMCU* significantly promotes the proliferation and metastasis of BC cells both in vitro and in vivo [52]. Mechanistically, binding sites for the let-7 family of miRNAs were found on *circHMCU*, which was validated by RNA immunoprepitation (RIP) and luciferase reporter assays [52]. The let-7 miRNA family is a well-known tumor suppressor and is responsible for the repression of several oncogenes, which include *MYC*, *HMGA2*, and *CCND1* [52]. Functional studies indicate that *circHMCU* acts as a miRNA sponge for let-7 and modulates BC proliferation and metastasis via regulating the expression of *MYC*, *HMGA2*, and *CCND1* [52].

***CircAGFG1***: *CircAGFG1* was found to be upregulated in TNBC tissues compared to matched adjacent normal tissues [67], and levels of the circRNA correlated with clinical stage, pathological grade, and poor prognosis of patients with TNBC [67]. Gain- and loss-of-function models using overexpression and RNA interference (RNAi) strategies indicated that *circAGFG1* promotes TNBC cell proliferation, mobility, and invasion in vitro as well as tumorigenesis and metastasis in vivo [67]. Potential targets of *circAGFG1* were identified using miRNA target prediction software and binding sites were found for miR-195-5p [67]. Interaction between *circAGFG1* and miR-195-5p was confirmed using an RNA-FISH assay in TNBC cells and tissues, and using luciferase reporter assays [67]. It was revealed that the mRNA of the cell cycle regulator CCNE1 also contains binding sites for miR-195-5p, and that knockdown (KD) of *circAGFG1* leads to decreased expression of CCNE1 in TNBC cells [67]. Thus, the findings from this study suggest that circAGFG1 might sponge miR-195-5p to modulate CCNE1 expression, leading to tumorigenesis and development of TNBC [67].

#### 3.1.2. Epithelial-to-Mesenchymal Transition (EMT)

***CircWWC3:*** Zinc finger E-box binding homeobox 1 (ZEB1) is a transcription factor and an important driver of BC progression [68]. Meng et al. attempted to identify ZEB1-regulated circRNAs in BC [69]. *CircWWC3* was one out of 15 identified circRNAs found to be upregulated following ZEB1 transfection in a TNBC cell line using a circRNA microarray [69]. ZEB1 was found to upregulate *circWWC3* expression but not the linear *WWC3* mRNA expression [69]. *WWC3* is a tumor suppressor that is downregulated in cancer and low *WWC3* expression is associated with poor prognosis of cancer patients [70]. Meng et al. demonstrated that linear *WWC3* mRNA levels were associated with good prognosis and inhibited BC cell growth and metastasis [69]. In contrast, elevated *circWWC3* levels were associated with poor prognosis in BC patients and exhibited oncogenic functions in BC [69]. These findings indicate that the competition of *circWWC3* with the linear *WWC3* mRNA promotes the progression of BC [69]. Silencing of *circWWC3* using siRNA significantly suppressed proliferation, migration, and invasion of BC cells [70]. Moreover, small hairpin RNA (shRNA)-mediated KD of *circWWC3* partially antagonized ZEB1-mediated BC growth and metastasis in vivo [69]. Mechanistically, *circWWC3* upregulates multiple oncogenes associated with the Ras signaling pathway by acting as a miRNA sponge for miR-26b-3p and miR-660-3p, as confirmed by luciferase reporter assays and RIP [69]. These oncogenes include *EGFR, GRB2, PAK4, MAPK1*, and *AKT1* [69]. Taken together, ZEB1-mediated upregulation of *circWWC3* promotes BC progression through activation of the Ras signaling pathway [69].

***CircANKS1B*:***CircANKS1B* was significantly upregulated in TNBC tissues compared to adjacent normal tissues as identified by RNA-seq [65]. Increased *circANKS1B* expression was associated with lymph node metastasis and advanced clinical stage, and served as an independent predictor of the overall survival of BC patients [65]. Functional studies revealed that *circANKS1B* promoted BC invasion and metastasis both in vitro and in vivo by inducing EMT, but had no effect on cell growth [65]. Mechanistically, *circANKS1B* abundantly sponged miR-148a-3p and miR-152-3p to increase the expression of the transcription factor USF1 [65]. USF1 upregulates TGF-β1 expression, resulting in activating TGF-β1/Smad signaling to promote EMT [65]. Additionally, Zeng et al. further demonstrated that *circANKS1B* biogenesis in BC was promoted by the splicing factor ESRP1, whose expression was also regulated by USF1 [65].

#### 3.1.3. Hypoxia

***CircDENND4C***: In order to identify hypoxia-associated circRNAs in BC, a panel of six previously reported hypoxia-associated circRNAs in endothelial cells was selected from published literature [71]. Hypoxia-inducible transcription factor alpha (HIF-1α) is a transcription factor that is activated under hypoxia, and high levels of HIF-1α correlate with poor prognosis in BC patients [72]. Induction of hypoxia in BC cells led to elevated levels of *circDENN4C*, and KD of HIF-1α reduced *circDENND4C* expression [71]. Although siRNA-mediated KD of *circDENN4C* resulted in reduced proliferation under hypoxic conditions, no phenotypic changes were observed under normoxia [71]. These findings indicate that *circDENND4C* manifests its function in regulating proliferation only in HIF1α-dependent-hypoxia, but not in normoxic environments [71]. In clinical specimens of BC, *circDENND4C* expression was more abundant in the tumor tissues than in adjacent non-cancerous tissues, and large-sized tumors showed increased *circDENND4C* expression levels compared to smaller-sized tumors [71]. A follow-up study using luciferase reporter assays and RIP to understand the underlying mechanism confirmed that *circDENN4C* acts as a miRNA sponge for miR-200b and miR-200c [73]. Rescue experiments revealed that KD of miR-200b and miR-200c attenuated the anti-cancer role of *circDENND4C* in BC under hypoxia, indicating that the circRNA regulates BC progression by sponging miR-200b and miR-200c [73].

***CircRNF20:*** Comparative RNA-seq of primary tumors and adjacent normal tissue revealed upregulation of *circRNF20* in BC [74]. Increased *circRNF20* levels are associated with poor clinical outcome in BC [74]. ShRNA KD of *circRNF20* repressed proliferation and resulted in reduced glucose uptake, lactate production, and ATP levels in BC cells [74]. RNA-FISH and luciferase reporter assays confirmed that *circRNF20* interacts with miR-487a, acting as miRNA sponge. This miRNA targets the 3′-UTR of HIF-1α. Moreover, HIF-1α binds to the promoter of the hexokinase II (HK2) gene and promotes its transcription [74]. In conclusion, this finding illustrates the vital role of *circRNF20* in BC progression and the Warburg effect [74]. 

#### 3.1.4. Autophagy

***CircDNMT1***: Increased expression of *circDNMT1* was identified in eight BC cell lines and in patients with BC using a microarray approach [75]. SiRNA-mediated silencing of *circDNMT1* inhibited cell proliferation and survival [75]. Ectopic expression of *circDNMT1* increased the proliferative and survival capacities of BC cells by stimulating cellular autophagy [75]. *CircDNMT1*-mediated autophagy led to inhibition of cellular senescence and increased tumor xenograft growth [75]. Ectopically expressed *circDNMT1* interacted with both p53 and AUF1 and promoted nuclear translocation of both proteins [75]. Nuclear translocation of p53 induced cellular autophagy, whereas AUF1 nuclear translocation reduced *DNMT1* mRNA instability and led to increased *DNMT1* translation [75]. Subsequently, it was found that functional DNMT1 translocated into the nucleus and inhibited p53 transcription [75]. Computational algorithms revealed that both p53 and AUF1 bind to different regions of *circDNMT1* [75]. Taken together, these findings indicate that highly expressed *circDNMT1* regulates oncogenic proteins in BC cells by direct interaction [75]. 

***CircCDYL:*** High-throughput sequencing of BC tissues with different autophagic levels led to identification of *circCDYL* [62]. Clinically, data from three independent cohorts showed a strong correlation of *circCDYL* expression levels and prognosis, as well as clinical response to therapy [62]. ShRNA-mediated KD of *circCDYL* demonstrated reduced proliferation, indicating that the circRNA promotes BC progression in vivo via autophagy [62]. Investigation into the mechanism of action was undertaken initially by analyzing for potential miRNA binding sites, followed by *circCDYL* pulldown and miRNA pulldown assays to confirm miR-1275 as a direct interacting partner of *circCDYL* [62]. Targets of miR-1275 include *ULK1* and *ATG7* mRNAs, which are associated with initiation of autophagy and autophagosome formation, respectively [62]. Subsequent experiments using miR-1275 inhibitors restored *ATG7* and *ULK1* expression after *circCDYL* KD, suggesting that this circRNA regulates the expression of the two genes by acting as a miR-1275 decoy [62].

#### 3.1.5. Invasion

***CircSKA3***: *CircSKA3* was identified as a highly expressed circRNA using microarray analysis of eight BC cell lines, and increased expression was further validated in BC patient samples compared to normal breast [76]. Loss- and gain-of-function experiments using siRNAs and *circSKA3* expression constructs indicated that *circSKA3* induced both invadopodium formation and cell invasion in gelatin degradation assays [76]. *CircSKA3* pull-down and immunoprecipitation of cell lysates revealed Tks5 and ITGB1 as direct binding partners [76]. Tks5 is required for the formation of invapodia and is a critical requirement for the invasiveness of BC cells [77]. ITGB1 is associated with enhanced features of cancer stem cells (CSCs), which include EMT, metastasis, and resistance to chemotherapy [78]. Follow-up experiments using cell fractionation and RNA-FISH combined with immunofluorescence validated the interaction between *circSKA3*, Tks5, and ITGB1 [76]. Furthermore, site-directed mutagenesis and immunoprecipitation confirmed putative binding sites for Tks5-ITGB1 on *circSKA3* that had previously been identified by bioinformatic analysis [76]. They further isolated *circSKA3*-Tks5-ITGB1 in invadopodium fractions and demonstrated that this circRNA-protein complex could be potentiating cell invasion [76].

Taken together, these studies suggest that circRNAs play a vital role in driving BC progression (summarized in Table 1) and indicate their potential as emerging therapeutic targets. 

### 3.2. CircRNAs as Tumor Suppressors

#### 3.2.1. Cell Cycle

***CircCCNB1***: Expression levels of different circRNAs in BC patients relative to the adjacent normal tissues were analyzed using a microarray, and expression of *circCCNB1* was found to be downregulated in cancer tissues [79]. CCNB1 is a regulator of mitosis, and high levels of CCNB1 are found in many cancers including BC [79]. Ectopic expression of *circCCNB1* decreased proliferation and survival, but increased apoptosis [79]. RIP assays using a probe targeting *circCCNB1* identified direct binding of H2AX and p53 in p53 wild-type cells [79]. Interestingly, similar studies using HTB126 BC cells, which carry a missense mutation for p53, revealed that *circCCNB1* formed a different complex, involving H2AX and BCLAF1 [79], a H2AX-dependent tumor suppressor [84]. Thus, this mechanism is specific to p53 mutant cells, as wild-type p53 has a greater affinity to H2AX, resulting in BCLAF1 binding to BCL2 instead and facilitating proliferation [79]. In the presence of mutant p53, BCLAF1 preferentially interacts with H2AX and *circCCNB1* to induce apoptosis [79].

A follow-up study revealed that this circRNA can also interact with CCNB1 and CDK1 [80]. In a normal cell, CCNB1 and CDK1 form a complex, allowing CCNB1 to function as an all-or-none switch for cell mitosis [85]. CCNB1 and CDK1 are both highly expressed in BC [86,87]. Cells transfected with *circCCNB1* followed by pull-down studies using *circCCNB1* probes revealed CDK1 and CCNB1 as its binding partners [80]. Cell fractionation studies further revealed that expression of *circCCNB1* decreased nuclear localization of CCNB1 and CDK1 [80]. The authors suggest that *circCCNB1* forms a complex with CCNB1 and CDK1, thereby preventing their translocation to the nucleus and suppressing the proliferation and survival of cancer cells [80]. In addition, ectopic expression of *circCCNB1* in vivo inhibited tumor growth and extended mouse viability [80]. However, these in vivo experiments were not performed with BC cells, but using B16 mouse melanoma cells stably transfected with *circCCNB1* and injected intraperitoneally [80]. Overall, these results have added to the role of *circCCNB1* in suppressing tumor progression in vitro and in vivo [80].

***CircFBXW7***: *CircFBXW7* was found downregulated in TNBC cell lines compared to normal tissue and other BC subtypes, and low expression of *circFBXW7* was associated with poor clinical outcome in 473 breast cancer patients [81]. Additionally, *circFBXW7* was negatively correlated with tumor size and lymph node metastasis, and it was an independent prognostic factor for TNBC patients [81]. Both in vitro and in vivo assays revealed that *circFBXW7* significantly suppressed cell proliferation and migration [81]. Mechanistic studies demonstrated that *circFBXW7* acts as a miRNA sponge for miR-197-3p to relieve the silencing of *FBXW7* [81]. FBXW7 is a crucial component of ubiquitin ligase and is considered a potent tumor suppressor, as most of its target substrates can function as potential growth promoters, including c-Myc, Notch, cyclin E, c-JUN, and KLF5 [88]. In addition, *circFBXW7* encodes the short protein FBXW7-185aa, which increases FBXW7 protein levels [81]. A previous study in glioma implicated that the short protein FBXW7-185aa interacts with the deubiquitinating enzyme USP28, preventing USP28 from binding to FBXW7 and antagonizing USP28-induced c-Myc stabilization [89]. Consistent with findings from the previous study [89], USP28 overexpression in BC cells reduced FBXW7 expression and suppressed FBXW7-185aa-induced c-Myc destabilization [81]. These findings indicate that *circFBXW7* upregulates FBXW7 expression two-fold: by removing miR-197-3p from the cellular pool, and via the small protein FBXW7-185aa, ultimately leading to suppression of TNBC progression [81].

#### 3.2.2. EMT

***CircKDM4C***: *CircKDM4C* was identified in a microarray as downregulated in metastatic BC compared to non-metastatic BC [64]. *CircKDM4C* levels were further examined in a cohort of BC patients and decreased expression was confirmed in BC samples compared to matched adjacent normal tissue [64]. Additionally, lower *circKDM4C* expression was associated with poor prognosis and metastasis in BC [64]. Functionally, siRNA-mediated KD of *circKDM4C* repressed BC proliferation, metastasis, and doxorubicin resistance in vitro and in vivo [64]. Mechanistically, it was identified as a miR-548p sponge using dual-luciferase activity assays and AGO2 RIP [64]. PBLD is a direct target of miR-548p, which functions as a tumor suppressor in BC [64]. PBLD was previously studied in hepatocellular carcinoma and was found to be a negative regulator of various tumor progression-related signaling pathways, such as MAPK signaling, EMT, and angiogenesis [90]. KD of PBLD increased proliferation of BC cells, further establishing the role of PBLD as a tumor suppressor in BC [64]. Moreover, miR-548p overexpression was able to reverse *circKDM4C*-induced attenuation of malignant phenotypes, and decreased expression of PBLD in BC cells [64].

#### 3.2.3. Invasion

***CircYAP***: *CircYAP* was identified as a negative regulator of YAP, a key component of the Hippo signaling pathway, which plays a crucial role in tumorigenesis [91]. Overexpression of *circYAP* in BC cells significantly decreased YAP protein expression, but did not affect *YAP* mRNA levels and suppressed the proliferation, migration, and colony formation of BC cells in vitro [82]. RIP and RNA pull-down assays indicated that *circYAP* binds to *YAP* mRNA and the translation initiation-associated proteins eIF4G and PABP [82]. Overexpression of *circYAP* abolished the interaction of PABP on the polyA tail with eIF4G on the 5’-cap of *YAP* mRNA, which led to the suppression of *YAP* translation initiation [82]. Individually blocking the binding sites of *circYAP* on *YAP* mRNA, or mutating the binding sites on *circYAP* for PABP and eIF4G induced *YAP* translation [82]. Dysregulation of *circYAP* expression was further confirmed in BC patient tissue [82]. This study uncovered a novel molecular mechanism in the regulation of YAP and implicated a new function of a circular RNA [82].

#### 3.2.4. Apoptosis

***CircFOXO3***: Of all circRNAs mentioned in this review, *circFOXO3* is the most well-characterized one. It has been associated with several cancers including BC, and its role in BC has been reviewed in detail previously [39]. *CircFOXO3* was initially found to be downregulated in BC cancer cells and patient tumor samples compared to adjacent normal tissue, but significantly upregulated in cancer cells during apoptosis [57]. SiRNA KD of *circFOXO3* showed enhanced cell viability and decreased apoptosis [57]. Investigation into the mechanism of action using RIP revealed that *circFOXO3* interacted with MDM2 and p53. Ectopic *circFOXO3* expression in vitro decreased p53 protein and increased FOXO3 and PUMA levels. Further pull-down experiments using a p53 antibody confirmed the presence of a *circFOXO3*-p53-MDM2 complex [57]. *CircFOXO3* facilitates p53 ubiquitination and degradation by directly binding to both p53 and MDM2 [57]. As a consequence, the capacity of MDM2 to induce ubiquitination and degradation of its other target FOXO3 is decreased, leading to increased stability of the FOXO3 protein. Increased FOXO3 levels then promote expression of PUMA and cell apoptosis [57].

#### 3.2.5. Immune Evasion

***CircTADA2A***: In a comparison of circRNA microarray data obtained from four TNBC and luminal BC samples, each with three normal mammary gland tissues, *circTADA2A-E6* was found to be significantly decreased, and low levels of this circRNA were linked to poor prognosis in BC patients [83]. *CircTADA2A-E6* suppressed in vitro cell proliferation, migration, invasion, and clonogenicity, indicating tumor-suppressor capability [83]. *CircTADA2A-E6* was described to act as a miR-203a-3p sponge, restoring the expression of the miR-203a-3p target gene *SOCS3*, a key regulator of cytokine signaling that inhibits BC proliferation [92], resulting in a less aggressive oncogenic phenotype [83]. 

The examples outlined above indicate that circRNAs can act as effective tumor suppressors in BC (summarized in Table 1). As mentioned previously, owing to their more stable nature [14], tumor-suppressive circRNAs could be ectopically expressed using adenoviral vectors as a way to combat tumor progression, and in this way could be used as cancer therapy [93]. However, in order for circRNAs to be used clinically, a more detailed understanding of their mechanism of action is essential and the development of safe delivery methods required.

## 4. Clinical Relevance of circRNAs in BC

Effective management of BC requires early diagnosis, reliable prognosis, and close monitoring of disease progression and therapy efficacy [94]. Liquid biopsies are a recently developed alternative to tissue biopsies for initial molecular diagnosis and management of tumor progression, especially throughout the course of treatment [95]. Currently, liquid biopsies are a rapidly expanding class of in vitro diagnostics (IVD) [95]. Nearly all types of molecular and cellular components in human blood have been explored as candidate targets for IVD development. These include circulating tumor cells, EVs, extracellular proteins, peptides, hormones, metabolites, extracellular DNA and their methylated and hydroxymethylated forms, and extracellular RNAs (exRNAs) [96]. A variety of exRNAs have been detected in human plasma and serum [95]. The repertoire of exRNAs found in biofluids also include long non-coding RNAs (lncRNAs) and circRNAs [97]. While miRNAs and lncRNAs have been studied extensively, circRNAs have not yet been explored in the context of BC to aid disease management and detect recurrence [25]. Due to their potentially higher stability, circRNAs may have an advantage over linear RNAs in regard to their application as biomarkers [97]. Although circRNAs as biomarkers in bodily fluids have been investigated in several types of cancers [25], they are a virtually untapped field in BC. More research is essential to exploit the role of extracellular circRNAs as diagnostic and therapeutic candidates in BC. Examples of some circRNAs with prognostic/diagnostic or therapeutic potential are detailed below.

### 4.1. CircRNAs as Diagnostic/Prognostic Markers

The majority of the studies on circRNAs associated with cancer focused on tissue biopsies and cell lines, with a wide range of data reported (as reviewed in [25]). A considerable number of published reports on potential circRNA biomarkers initially identified circRNAs dysregulated in cancer tissue, and further investigation revealed that the same signature was mirrored in bodily fluids [25]. One study demonstrated the prognostic potential of the circRNA *hsa_circ_0001785* by analyzing circRNA expression profiles in BC peripheral blood [98]. The expression levels of *hsa_circ_0001785* was found to have decreased in the plasma samples of post-operative BC patients compared to pre-operative patients [98]. However, the underlying mechanism of *hsa_circ_0001785* has not been studied thus far, and it remains elusive whether this circRNA is directly involved in driving BC progression.

Additional examples of circRNAs with potential as biomarkers include some of the above discussed circRNAs. For instance, *circHMCU*, *circDNMT1*, *circDENND4C*, *circRNF20*, and *circSKA3* may prove to be useful as general diagnostic markers for BC, as their expression is upregulated regardless of BC subtype. Similarly, *circAGFG1* and *circANKS1B* may be suitable biomarkers in TNBC, as their expression levels are TNBC-specific. Of these, levels of *circAGFG1*, *circCDYL*, and *circANKS1B* correlated with histological grade, TNM stage, and distant metastasis, which may aid in the staging and grading of BC. Interestingly, none of the circRNAs discussed here have been investigated as candidate biomarkers for liquid biopsy thus far. We suggest that there is a vast untapped potential for circRNAs as diagnostic/prognostic biomarkers in BC.

### 4.2. CircRNAs as Markers of Drug Resistance

Chemotherapy is an effective method to prevent BC recurrence and metastasis following surgical treatment [99]. However, chemotherapeutic resistance remains a major problem, and a way to track or predict the development of drug resistance would enable better management of BC [100]. To this end, some circRNAs were described as associated with drug resistance (Section 3.2.2), and therefore may be relevant as biomarkers in this space [99]. The mechanism of action in drug resistance has been studied for a number of circRNAs in other types of cancer previously [101,102,103], but only a handful of candidates were investigated in detail in BC. One example of a circRNA involved in drug resistance in BC is the above discussed *circKDM4C* [64]. Downregulation of *circKDM4C* inhibits tumor progression and attenuates doxorubicin resistance by acting as a miRNA sponge for miR-548p [64], indicating its potential as a biomarker for doxorubicin resistance.

### 4.3. CircRNAs as Therapies and Therapeutic Targets in BC

Recent advances in RNA-based therapeutics coupled with aberrant expression of circRNAs in BC makes them attractive therapeutic targets [104]. One possible approach is the design of synthetic circRNAs with multiple binding sites for specific oncogenic proteins or miRNAs, which could be introduced exogenously to restore the normal regulatory network in the cell and limit cancer growth [43,105].

Alternatively, endogenous circRNAs may be suitable as cancer therapies [106]. Some of the tumor-suppressor circRNAs reviewed above are potential candidates to be exploited further as therapeutic tools. *CircFOXO3*, *circCCNB1*, *circKDM4C*, *circFBXW*, and *circTADA2A* were all downregulated in patient samples, associated with poor prognosis, and are functionally linked to cancer etiology. A therapeutic approach may involve using circRNA overexpression constructs delivered via adeno-associated virus (AAV) vectors, as these vectors do not integrate into the genome and are currently used in clinical trials [107].

On the other hand, oncogenic circRNAs, including the TNBC-specific *circAGFG1* and *circANKS1B*, may be exploited as novel therapeutic targets for TNBC, which currently lacks extensive treatment options and is associated with poor prognosis [7]. Multiple strategies have been developed to target overexpressed circRNA expression therapeutically, such as degradation mediated via siRNA, shRNA, or modified antisense oligonucleotides (ASOs) complementary to the back-splice junction [108,109]. More stable knockout strategies have also been investigated, including CRISPR/Cas genome editing. A recent addition is the CRISPR/Cas13 system wherein circRNA silencing is attained by targeting Cas13 to the back-splice junction of the circRNA via a specific guide RNA, which is able to distinguish between linear transcripts and circRNAs [110]. In particular, Cas13d, a small variant of Cas13 [111] can possibly be packaged into an AAV vector for delivery into primary cells and mice [112]. However, use of this system in a therapeutic context may not be applicable yet as side effects of Cas13 expression are currently unknown [110].

## 5. Limitations and Challenges in the Field of CircRNAs in BC

Recently, a lot of research has been undertaken in the field of circRNAs in BC [62,71,75,76]. The majority of published research focuses on the discovery of circRNAs in BC, and functional studies have not been accomplished for most of these molecules [66]. On the path to circRNA discovery, a considerable number of studies have used publicly available datasets such as The Cancer Genome Atlas (TCGA) data [113], which may not be ideal as these RNA-seq libraries were generated using polyA enrichment, thus potentially omitting numerous circRNAs [114]. Generating new datasets specifically aimed at circRNA discovery is essential to annotating the complement of circRNAs in the future.

With regards to the functional roles of circRNAs, investigating the sub-cellular localization of circRNAs is crucial, as this will provide insights into their possible molecular mechanism. While RNA-FISH is an excellent method to determine sub-cellular localization for non-coding RNAs in general, it may be challenging to specifically design tiling sets of probes for some circRNAs due to the limitation of probe design targeting the back-spliced junction [115]. Similarly, limited options to design specific probes generate challenges for pull-down experiments of circRNAs, in particular if the circRNA of interest is also of low abundance in the cell. Increasing sensitivity of detection and single-molecule methods could alleviate these challenges in the future.

While the mechanism of action has been described for some circRNAs in BC, most studies focused on investigating circRNAs as miRNA sponges [63,67,116]. Thus far, few studies have shifted their attention to other potential circRNA interacting partners, such as other classes of RNA or proteins [75]. A detailed understanding of how circRNAs function is still lacking, which is in part due to the lack of robust techniques to study this type of transcript [115].

Nevertheless, circRNAs are being exploited as viable biomarkers in other cancers [117,118] and a clinical trial is currently underway to identify circRNA biomarkers in plasma samples of patients with pancreaticobiliary cancer (clinical trial identifier: NCT04584996) [119]. Thus far, most studies are limited to tissues, and the presence of circRNAs in bodily fluids needs to be investigated further to examine their use as diagnostic/prognostic markers in BC. For circRNAs to be used as therapies or therapeutic targets, safe delivery methods and effective KD strategies need to be developed.

## 6. Conclusions

Expression levels of specific circRNAs have been demonstrated to correlate with BC progression and subtype, with a number of circRNAs being associated with poor prognosis. Functionally, circRNAs have been found to regulate BC progression by impacting cancer cell proliferation, migration, invasion, and metastasis, and a few seem to be involved in drug resistance. From a mechanistic standpoint, most circRNAs studied thus far seem to act as molecular decoy, most often for miRNA. The enhanced stability of circRNAs together with their presence in bodily fluids will enable their detection in a clinical setting in the future. Overall, circRNAs are an emerging class of potential biomarkers and new therapeutic targets in BC.

## Figures and Tables

**Table 1:** CircRNAs involved in breast cancer progression.

CircRNA	Expression in BC	Function in BC	Mechanism of Action	Reference
*CircHMCU*	Upregulated	Promotes proliferation and metastasis	Modulates MYC, HMGA2, and CCND1 expression by acting as a miRNA sponge of let-7	[52]
*CircAGFG1*	Upregulated	Promotes proliferation, invasion, and metastasis	Regulates CCNE1 expression by acting as a sponge for miR-195-5p	[67]
*CircWWC3*	Upregulated	Promotes epithelial-to-mesenchymal transition (EMT)	Acts as a sponge for miR-26b-3p and miR-660-3p and upregulates expression of EGFR, GRB2, PAK4, MAPK1, and AKT1 (Ras signaling pathway)	[69]
*CircANKS1B*	Upregulated	Induces EMT	Sponges miR-148-3p and miR-152-3p and increases expression of USF1. USF1 upregulates TGF-β1	[65]
*CircDENND4C*	Upregulated	Regulates proliferation under hypoxic condition	Acts as a sponge for miR-200b and miR-200c under hypoxia	[71,73]
*CircRNF20*	Upregulated	Increases glucose uptake and lactate production	Sponges miR-487a, which targets 3′ UTR of HIF1 α	[74]
*CircDNMT1*	Upregulated	Stimulates cellular autophagy	Interacts with p53 and AUF1 and promotes their nuclear translocation, which induces autophagy and reduces DNMT1 mRNA stability	[75]
*CircCDYL*	Upregulated	Promotes proliferation via autophagy	Acts as a miR-1275 decoy, which targets ULK1 and ATG7 mRNAs associated with autophagy and autophagosome	[62]
*CircSKA3*	Upregulated	Induces invapodium formation and cell invasion	Interacts with Tks5 and ITGB1 CircSKA3-Tks5-ITGB1 complex promotes cell invasion	[76]
*CircCCNB1*	Downregulated	Decreases cell proliferation and survival, increases apoptosis	In mutant p53 cells, BCLAF1 interacts with H2AX and circCCNB1 to induce apoptosis Forms a complex with CCNB1 and CDK1 and prevents nuclear translocation, thereby suppressing cell proliferation and survival	[79,80]
*CircFBXW7*	Downregulated	Suppresses cell proliferation and migration	Upregulates FBWX7 expression by sponging miR-197-3p	[81]
*circKDM4C*	Downregulated	Suppresses proliferation, metastasis, and doxorubicin resistance	Sponges miR-548p, which targets a tumor-suppressor PBLD	[64]
*CircYAP*	Downregulated	Supresses proliferation and migration	Inhibits YAP translation initiation by interacting with eIF4G and PABP	[82]
*CircFOXO3*	Downregulated	Decreases cell viability and increases apoptosis	Facilitates p53 ubiquitination and degration by binding to p53 and MDM2. Leads to increased stability of FOXO3 which promotes PUMA expression and cell apoptosis	[57]
*CircTADA2A*	Downregulated	Suppresses proliferation, migration, invasion, and clonogenicity	Sponges miR-203a-3p, which leads to increased expression of SOCS3, regulator of cytokine signaling	[83]

## Data Availability

Not applicable.
